# Plant Polyphenols-Biofortified Foods as a Novel Tool for the Prevention of Human Gut Diseases

**DOI:** 10.3390/antiox9121225

**Published:** 2020-12-03

**Authors:** Aurelia Scarano, Marcello Chieppa, Angelo Santino

**Affiliations:** 1 Institute of Science of Food Production, C.N.R. Unit of Lecce, 73100 Lecce, Italy; aurelia.scarano@ispa.cnr.it; 2 National Institute of Gastroenterology ‘S. De Bellis’, Institute of Research, 70013 Castellana Grotte, Italy; marcello.chieppa@irccsdebellis.it

**Keywords:** plant polyphenols, gut health, biotechnology, biofortification

## Abstract

Plant food biofortification is recently receiving remarkable attention, as it aims to increase the intake of minerals, vitamins, or antioxidants, crucial for their contribution to the general human health status and disease prevention. In this context, the study of the plant’s secondary metabolites, such as polyphenols, plays a pivotal role for the development of a new generation of plant crops, compensating, at least in part, the low nutritional quality of Western diets with a higher quality of dietary sources. Due to the prevalent immunomodulatory activity at the intestinal level, polyphenols represent a nutritionally relevant class of plant secondary metabolites. In this review, we focus on the antioxidant and anti-inflammatory properties of different classes of polyphenols with a specific attention to their potential in the prevention of intestinal pathological processes. We also discuss the latest biotechnology strategies and new advances of genomic techniques as a helpful tool for polyphenols biofortification and the development of novel, healthy dietary alternatives that can contribute to the prevention of inflammatory bowel diseases.

## 1. The Polyphenols: Key Specialized Metabolites for Plants

Polyphenols are secondary metabolites, widespread in many fruits, vegetables and plant byproducts commonly consumed in the human diet. Examples of dietary sources rich in polyphenols include berries (i.e., blueberries, blackberries, black currants, etc.), apples, citrus species, grapes, coffee, tea, cocoa or vegetable crops, such as onions, carrots, artichokes, solanaceous species, and cruciferous plants (Table 1) [1]. Other less common under-utilized species rich in polyphenols include, for example, figs, white crowberry, glasswort, and gooseberries, which are recently attracting more attention as alternative dietary patterns enriched in healthy beneficial phytochemicals (Table 1).

Polyphenols are one of the most widespread and studied group of plant secondary metabolites, mainly for their contribution to the nutritional value of plant food-based diets and their benefits for human health. The consumption of foods and beverages enriched in polyphenols have been associated to the prevention of human diseases related to cellular oxidative stress [2]. Beside the well-known antioxidant activities, polyphenols have also investigated for anti-inflammatory properties at molecular level and protection against aging and chronic diseases, such as inflammatory bowel diseases [2,3].

Plant polyphenols show a wide structural and chemical variability, among different families of plant kingdom or within the same family [4]. This structural diversity suggests that they do not play the same function: the different biological activities are influenced by the amounts and the nature of the lateral moieties linked to the basic structural backbone, composed by variously hydroxylated or decorated aromatic rings. The presence of hydroxyl groups, for example, confers to polyphenols the capacity to participate in oxidation reactions, which take part in several physiological processes [5]. Some polyphenols are pigmented (i.e., anthocyanins), therefore, being involved in the attraction of pollinators and seed-dispersers [6]. Other polyphenols are involved in the processes of cellular growth (i.e., hydroxycinnamic acids, lignins). Phenolic acids are directly involved in the defense towards different stresses, since they contribute to lignification of wounded tissues and have antimicrobial activities [7]. Flavonoids, one of the most common and studied classes of polyphenols, function as sunscreens and protect from UV exposure [8,9].

**Figure 1:**
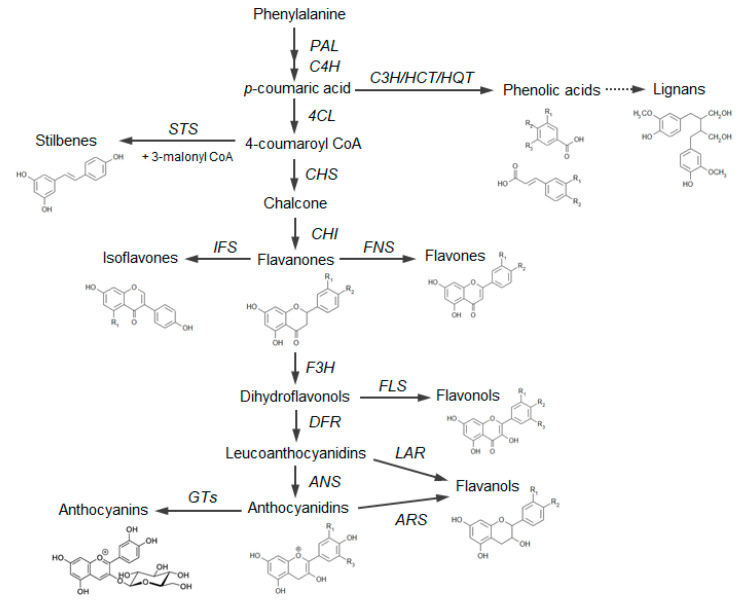
Schematic representation of the general polyphenol biosynthetic pathway, with the main classes of polyphenols. PAL, Phenylalanine ammonia lyase; C4H, cinnamic acid 4-hydroxylase; C3H, p-coumarate 3-hydroxylase; HCT, hydroxycinnamoyl CoA shikimate/quinate hydroxycinnamoyl transferase; HQT, hydroxycinnamoyl CoA quinate hydroxycinnamoyl transferase; 4CL, 4-coumarate:coenzyme A ligase; STS, stilbene synthase; CHS, chalcone synthase; CHI, chalcone isomerase; IFS, isoflavone synthase; FNS, flavone synthase; F3H, flavanone-3-hydroxylase; FLS, flavonol synthase; DFR, dihydroflavonol reductase; LAR, leucoanthocyanidin reductase; ANS, anthocyanidin synthase; ARS, anthocyanidin reductase; GTs, glycosyltransferases.

Polyphenols are synthesized in plants via phenylpropanoid pathway (Figure 1), through a finely orchestrated sequence of biochemical steps, coordinated by different structural and regulatory genes. Hence, the variability in the quantity and type of polyphenols found in a specific plant species derives from the activation of these genes resulting from the genetic background and plant environmental adaptation [10,11].

Based on the number of aromatic rings and the structural elements linked to these basic rings, polyphenols can be distinguished in different classes: phenolic acids, including hydroxybenzoic and hydroxycinnamic acids; flavonoids, including flavonols, flavanones, isoflavones, flavanols; anthocyanins; stilbenes [12] (Figure 1; Table 1).

**Table 1:** Main polyphenol classes, content in dietary sources and detection methods. TCPA: total content of phenolic acids; TCF: total content of flavonoids; TCA, total content of anthocyanidins; TCS: total content of stilbenes; TPC: total polyphenol content.

Polyphenol Class	Dietary Source and Content	Method of Determination	Reference
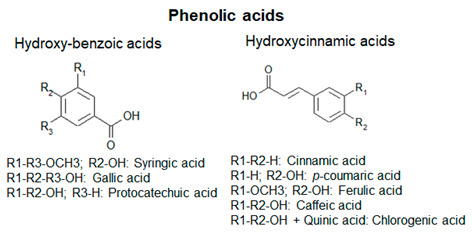	Carrots (TCPA: 0.3–18.8 g·kg^−1^ DW; 0.5 g·kg^−1^ FW)	Spectrophotometric (Folin-Ciocalteau)/ RP-HPLC DAD	[13,14]
	Artichokes (TCPA: 3.14–3.89 g·kg^−1^ DW)	RP-HPLC DAD	[15,16]
	Coffee (TCPA: 5 g·kg^−1^ coffee pulp)	RP-HPLC UV/VIS	[17]
	White crowberry (TPC: 16.140 g GAE·kg^−1^ DW)	Spectrophotometric (Folin-Ciocalteau)	[18]
	Glasswort (TPC: 290.5 mg GAE·kg^−1^ FW)	Spectrophotometric (Folin-Ciocalteau)	[19]
	Lettuce (TPC: 127–187 mg GAE·kg^−1^ FW)	Spectrophotometric/HPLC-DAD- ESI/MSn	[20]
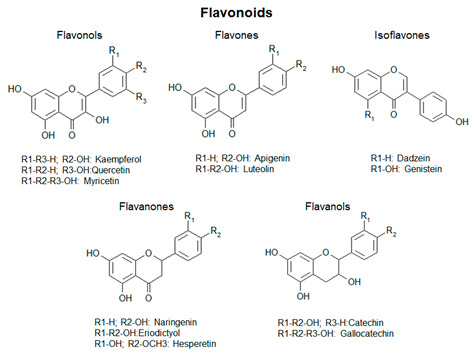	Onions (flavonols: 81–93 mg·kg^−1^ FW)	RP-HPLC PDA	[21]
	Leek (flavonols: 160 mg·kg^−1^ FW)	RP-HPLC PDA	[21]
	Tomato (flavonols: 68–105 mg·kg^−1^ FW)	RP-HPLC PDA	[21]
	Broccoli (flavonols: 2 mg·kg^−1^ FW; 6.75 g·kg^−1^ FW)	Spectrophotometric RP-HPLC UV	[22,23]
	Kale (flavonols: 115 mg·kg^−1^ FW)	RP-HPLC PDA	[21]
	Cauliflower (flavonols: 37.53 g·kg^−1^ FW)	RP-HPLC UV	[23]
	Soybean (flavones: 9.4 mg·kg^−1^ FW)	RP-HPLC PDA	[21]
	Soybean (isoflavones: 0.5–7.5 mg·kg^−1^ FW)	RP-HPLC DAD	[24]
	Citrus fruit (flavanones: 98–4694 mg·kg^−1^ FW)	RP-HPLC PDA	[25]
	Apricots (flavan-3-ols: 250 mg·kg^−1^ FW)	RP-HPLC PDA	[21]
	Green Tea (brew) (TCF: 8.30 g QE·kg^−1^ DW; flavan-3-ols: 350–441 mg·100^−1^ mL; 12.81–43.65 g·kg^−1^ DW; TPC: 68.3–85.9 mg·100^−1^ mL)	RP-HPLC DAD/ LC-ESI-Q-TOF-MS/ Spectrophotometric (Folin-Ciocalteau)	[26,27,28]
	Black Tea (brew) (TCF: 7 g QE·kg^−1^ DW)	RP-HPLC PDA/ Spectrophotometric	[26]
	Apples (flavonols: 300–344 mg·kg^−1^ FW)	RP-HPLC PDA	[21]
	Blueberries (flavonols: 172–327 mg·kg^−1^ FW)	HPLC/ESI-MS	[29]
	Grapes (flavonols: 21–322 mg·kg^−1^ FW)	HPLC/ESI-MS	[29]
	Cocoa (flavan-3-ols: 45–730 mg·kg^−1^ DW; (flavonols: 0.3–42 mg·kg^−1^ DW)	LC-DAD, LC-MS	[30]
	Figs (flavan-3-ols: 29.6–130.2 g·kg^−1^ DW; (flavonols: 79.9–21.78 g·kg^−1^ DW)	UPLC-PDA-FL	[31]
	Gooseberry (TCF: 3.04–6.99 g RE·kg^−1^ DW; 345.0–3726.5 mg·kg^−1^ DW)	RP-HPLC DAD/ Spectrophotometric	[32]
	Currant (TCF: 8.13–15.62 g RE·kg^−1^)c	RP-HPLC DAD/ Spectrophotometric	[32]
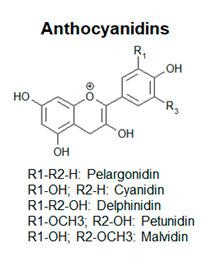	Red cabbage (TCA: 40–750 mg·kg^−1^ FW)	HPLC-MS/MS	[33]
	Black carrots (TCA: 14–177 mg·kg^−1^ FW)	RP-HPLC PDA/HPLC/ESI-MS/	[34]
	Blueberries (TCA: 1435–8227 mg·kg^−1^ FW)	Spectrophotometric HPLC/ESI-MS	[29]
	Blackberries (TCA: 2–4 g·kg^−1^ FW)	HPLC/ESI-MS	[29,35]
	Grapes (TCA: 390–7900 mg·kg^−1^ FW)	HPLC/ESI-MS	[29]
	Beetroot (TCA: 0.23–0.77 g Mv3G·kg^−1^ FW)	Spectrophotometric (Folin-Ciocalteau)	[36]
	Strawberries (TCA: 180 mg Mv3G·kg^−1^ FW)	Spectrophotometric (Folin-Ciocalteau)	[37]
	Figs (TCA: 4–1220 mg·kg^−1^ DW)	UPLC-PDA-FL	[31]
	Gooseberry (TCA: 0.3–686.8 mg COG·kg^−1^ DW)	RP-HPLC DAD/ Spectrophotometric	[32]
	Currant (TCA: 603.4–1407.1 mg COG·kg^−1^ DW)	RP-HPLC DAD/ Spectrophotometric	[32]
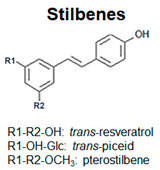	Grapes (TCS: 0.1–1.7 g·kg^−1^ FW)	RP-HPLC UV	[38]
	Red currant (TCS: 15 mg·kg^−1^ FW)	Capillary electrophoresis	[39]
	Blueberries (TCS: 4 mg·kg^−1^ FW)	LC-MS	[39]
	Peanuts (TCS: 0.01–15 mg·kg^−1^ FW)	RP-HPLC DAD	[40]

## 2. Plant Polyphenols and Gut Health: A Focus on the Antioxidant and Anti-Inflammatory Properties

Plant polyphenols commonly assumed with fruits and vegetables in human diet have been extensively associated to healthy beneficial effects, ranging from the antiaging, anti-tumoral, or anti-diabetic effects, to a positive impact on non-communicable diseases, such as cardiovascular or neurodegenerative diseases [1,41]. Several studies have also evidenced a beneficial impact of polyphenols for a healthy intestinal status, contributing to the prevention and protection against gut pathological processes, which often evolve in both acute and chronic states of inflammation leading to inflammatory bowel diseases (IBDs). Such pathologic conditions concur to stimulate the immune system at gut and systemic level [42]. In this context, nutritional strategies, including dietary anti-inflammatory compounds, could be beneficial at least in the prevention of these diseases [43,44].

Polyphenols are one of the most abundant antioxidant class in human diet [45]. A first reason explaining polyphenols antioxidant activities lies on their chemical structure and essentially on the presence of phenolic groups, particularly cathecol or galloyl groups [46]. Most of polyphenols are reducing agents and together with ascorbic acid, carotenoids and vitamin E, contribute to cell protection from tissue oxidative stress and damage [47]. Being reducing agents, polyphenols can exert a direct antioxidant activity on free radicals and Reactive Oxygen Species (ROS). Besides this “scavenging” role, polyphenols can have direct effects on oxidation of membrane lipids and the modulation of different enzymes [47,48], such as glutathione peroxidase and superoxide dismutase [41,48]. Through direct interaction with proteins involved in signal transduction, polyphenols can influence the cell redox status and trigger a series of redox-dependent reactions [45]. For example, Moskaug and co-workers [49] showed that flavonoids are able to increase the expression of γ-glutamylcisteine synthase (a rate-limiting enzyme in the synthesis of glutathione), with a concomitant increase of glutathione. As glutathione is implicated in several physiological and pathological cellular processes, polyphenols-mediated regulation of glutathione concentration can be one mechanism by which diet can influence oxidative related diseases [49].

Polyphenols can also impact on immune responses and intracellular signaling pathways, especially at intestinal level [3,50,51,52]. The effects on the inflammatory responses involve both cellular (macrophages, dendritic cells, lymphocytes) and molecular (cytokines, interleukins) mediators of inflammation. In vitro and in vivo studies have clearly demonstrated the anti-inflammatory effects of polyphenols (see Table 2), and their metabolites deriving from the processes of biotransformation at gut microbiota level [53,54,55,56].

A first anti-inflammatory mechanism concerns the modulation of mediators of inflammation through the phospholipase A2 and cyclo-oxygenase 2 (COX-2) enzymatic activities. Polyphenols have been reported to reduce COX-2 activity and relative mRNAs in different cell lines [57,58]. A second mechanism involves the modulation of cytokines. For example, flavonoids are able to impair the expression of the interleukins IL-1β, IL-6, IL-8, and tumor necrosis factor (TNF) [51,53]. Other signaling pathways modulated by polyphenols participate to the inflammatory responses, proliferation and apoptosis, as in the case of the NFkB signaling pathway (Nuclear Factor kappa-light-chain enhancer of activated B cells [59]), and the MAP kinases pathway (Mitogen-Activated Protein kinase; [3,60]). In fact, the convergence of oxidative stress, inflammatory stimuli and their amplification by inflammatory cells and cytokines, culminate with the IkB kinases (IkKs) activation and the consequent IkB (cytoplasmic inhibitor of kB) phosphorylation.

NFkB, which is normally retained by IkB in the inactive form, is released after IkB phosphorylation, and once translocated to the nucleus, it induces the transcription of inflammatory genes (e.g., COX-2, TNFα, inducible nitric oxide synthase - iNOS). Polyphenols like epigallocatechin gallate, quercetin and anthocyanins have been showed to inhibit IkK phosphorylation, with consequent inhibition of the NFkB activation [56,61].

In the case of the MAPKs signaling pathway, polyphenols like anthocyanins can modulate the MAPK phosphorylation (p38MAPK, ERK, JNK) or the activity of transcription factors targeted by MAPKs (e.g., STAT-1, STAT-3) [3,61].

Another important mechanism concerns the nitric oxide (NO) production at the vascular level. NO is an essential component for vascular health maintenance and is involved in the inflammatory response triggered by free radicals. Some studies suggest that polyphenols inhibit NO release, suppressing the expression of endothelial nitric oxide synthetase (eNOS) [62,63]. Polyphenols can also promote a vascular remodeling activity and inhibit the extracellular matrix-degrading enzymes, i.e., the matrix metalloproteinase-2 and -9 [64,65].

Yet, an emerging and recently proposed anti-inflammatory mechanism is related to the cellular iron homeostasis and metabolism of dendritic cells (DCs), one of the major players of the intestinal adaptive immune response. The balance between the iron intracellular efflux and its extracellular transport (import/export) has been associated to DCs activation [66]. Iron uptake is a crucial response for immune cells against invading bacteria, whereas a physiological status of tolerance and tissue repair favors iron degradation and release, mediated by heme-oxygenase 1 (Hmox-1) and Ferroportin-1 [67]. The administration of iron-sequestrating quercetin, for example, induces a cascade of events starting with a strong up-regulation of Hmox-1 and Ferroportin-1 in DCs, resulting in iron release in the extracellular compartment and a reduction of cytoplasmic iron content [66]. Iron-depleted immune cells fail to produce inflammatory mediators while supporting the release of secretory leukoprotease inhibitor (Slpi) [68,69].

The preventive role of polyphenols on gut inflammation has been further confirmed using in vivo models. To date, several studies on rodent models of experimentally-induced IBDs, have indicated that dietary polyphenols can be effective in the prevention of these idiopathic diseases [70]. For example, the oral supplementation of dried bilberries reduced the pathological signs of inflammation, including IFNγ and TNF production by mesenteric lymph node cells, in a Balb/c mouse model of acute colitis induced by dextran sodium sulfate (DSS) [71]. The administration of pomegranate extract and its ellagic acid rich fraction has been described with anti-ulcerative effects and amelioration of the inflammatory condition in DSS-induced colitis [72]. In the same murine model, the administration of white or red grape-based diets, as well as of an engineered polyphenol-enriched tomato diet, has been useful to reduce inflammation symptoms and decrease the pro-inflammatory IL-6 and TNF [73]. Confirming results have been described in a spontaneously developing colitis mouse model (Winnie mice), with preventive roles under both homeostatic or inflammatory conditions [74].

Together with a direct anti-inflammatory role displayed by polyphenols, an increasing amount of literature is more recently exploring the possibility that they can favor the growth of beneficial microbial communities [75,76,77]. Polyphenol-induced changes on the gut microbiota have been described either at phylum and genera levels, modifications that could be taken into consideration in the treatment of intestinal dysbiosis, which can trigger or exacerbate a chronic inflammation status. For example, the dietary administration of blueberries, blackcurrants and other sources containing polyphenols, have been reported to beneficially impact on the *Firmicutes*/*Bacteroidetes* ratio in murine models [73,74,78]. The promotion of the growth of probiotic genera, such as *Bifidobacterium* or *Lactobacillus*, has been observed following anthocyanins-enriched diets [73,79]. On the other hand, antimicrobial effects and a reduction in the growth of *Bacteroides*, *Prevotella*, *Oscillospira*, *Blautia* genera and *Clostridium* spp. (these latest often associated to pathological conditions) have also been described following polyphenol administration [53,78,80,81,82]. However, an important aspect that should be considered is the bioavailability and the consequent bio-efficacy of polyphenols, which is largely influenced by the physicochemical properties of polyphenols, the modifications they undergo at gut level, the composition and interactions with complex food matrices and the biotransformation processes exerted by microbial groups, converting polyphenols in more simple, absorbable metabolites [83]. The bio-efficacy of microbiota metabolized forms of polyphenols have also been studied [53,54], highlighting that these forms can also display relevant biological properties [75].

Taken together all these findings indicate an overall bioactive potential of polyphenols useful for the prevention of inflammatory conditions at the gut level and point the central issue related to their content in the main fruits and vegetables commonly consumed by Wstern people.

**Table 2:** Examples of in vitro and in vivo studies used to evaluate polyphenols antioxidant and anti-inflammatory activities at intestinal level.

Plant Sources and Class of Polyphenols	In Vitro/In Vivo Model	Type of Administration/ Treatment	Biological Activity	Reference
White grape skin (phenolic acids, proanthocyanidin, catechin, quercetin)	HT-29 cells	Methanolic extract	Restored stress-related GSH reduction by polyphenols in intestinal cells	[84]
Apple (catechins, chlorogenic acid)	MKN 28 cells; Male Wistar rats	Methanolic extract; polyphenol administration by drinking water or gavage	Prevention of oxidative injury in gastric epithelial cells and gastric mucosa	[85]
Apple (flavonoids, phenolic acids)	HT-29 cells; CaCo-2 cells	Cider, apple juice	High preventive antioxidant capacity, decreased cellular reactive oxygen species, reduced oxidative cell damage	[86]
Grape pomace (phenolic acids, procyanidins)	IPEC-1 cells; TOPIG hybrid pigs	Aqueous extract; dietary administration of grape pomace	Decrease of lipid peroxidation in duodenum and colon and increase of the total antioxidant status	[87]
Pomegranate (ellagitannins)	Liposome model (large unilamellar vesicles, LUVs)	Pomegranate juice	Inhibition of the lipid peroxidation	[88]
Blueberries (anthocyanins)	CaCo-2 cells	Methanolic extracts	Reduced cellular oxidative stress	[89]
Red wine (tannins; anthocyanins)	HT-29 cells	Extract from red wine	Reduced iNOS and COX-2 levels, modulation of the NFkB signaling pathway	[90]
Grapeseeds (flavonoids)	CaCo-2 cells	Ethanolic extract	Reduced NfKB transactivation and TNFα transcripts levels	[91]
Dried peel of apple (flavonols, catechins, procyanidins)	CaCo-2/15 cells	Crude extract and purified polyphenolic fraction	TNFα, IL6, E2 prostaglandin, COX2, NFkB down-regulation	[92]
Purple engineered tomato (flavonoids, anthocyanins)	CEC cells	Methanolic extracts	Inhibition of the pro-inflammatory cytokines	[93]
Green tea (polyphenols)	BALB/c mice with DSS-induced colitis	Dietary administration in chow diet	Reduction of TNFα and GSH levels	[94]
Strawberries (anthocyanins)	Wistar rats with ethanol-induced gastric lesions	Oral administration of raw extract	Reduction of the ulcerative index; reduction of the gastric lipid peroxidation	[95]
Apple polyphenols	C57/BL6 mice with DSS-induced colitis	Administration of polyphenol mix in drinking water or oral gavage	Reduced levels of mediators of inflammation (TNFα, IFNγ, IL1β, IL6, IL17, IL22)	[96]
Green tea polyphenols (epigallocatechin-3-gallate)	C57/BL6 mice with DSS-induced colitis	Administration of polyphenol mix via oral gavage	Reduction of tissue damage and neutrophiles accumulation; increased levels of antioxidant enzymes	[97]
Grape juice	Wistar rats with TNBS-induced colitis	Administration in drinking water	Reduced inflammatory activity	[98]
Grape pomace	Wistar rats with DSS-induced colitis	Administration in chow diet	Reduced tissue damage and pro-inflammatory cytokines levels	[99]
Cocoa polyphenols	BALB/c mice with DSS-induced colitis	Administration in chow diet	Reduction of tumoral incidence, partially limited activation of the IL-6/STAT3 pathway	[100]
Bronze engineered tomato (flavonols, anthocyanins, stilbenes)	C57/BL6 mice with DSS-induced colitis	Administration in chow diet	Reduced inflammatory symptoms; beneficial changes in gut microbiota composition, reduced pro-inflammatory cytokines levels	[73]
Red and white grape skin (flavonols, anthocyanins, stilbenes)	C57/BL6 mice with DSS-induced colitis	Administration in chow diet	Reduced inflammatory symptoms; reduced pro-inflammatory cytokines levels	[73]
Bronze engineered tomato (flavonols, anthocyanins, stilbenes)	Winnie mice spontaneously developing colitis	Administration in chow diet	Changes in gut microbiota composition; reduced pro-inflammatory cytokines levels	[74]

## 3. Biofortification Strategies for the Improvement of Polyphenol Content in Plant Food

Daily intake of polyphenols is considered suboptimal in many Western countries. Del Bo’ and co-workers [101] suggested a total polyphenol intake of about 900 mg/day, considering literature studies on polyphenol intake assessment using various quantification methodologies and some polyphenols databases sources (e.g., USDA, Phenol-Explorer). A large variation in polyphenols uptake was also observed according different dietary patterns (e.g., Mediterranean diet, Western diet), lifestyle, educational and economic levels, significantly affecting the general eating habits.

Therefore, people are asked to shift their nutritional habits towards a larger use of fruits and vegetables. However, as indicated in Table 1, many species where higher amounts of polyphenols have been reported are not largely consumed or available throughout all the year. Furthermore, a large amount of bioactive compounds are lost after cooking, food processing phases or other common practices frequently undertaken before ingestion (i.e., peel removal). Therefore, the increase in the content of healthy beneficial plant secondary metabolites is mandatory to address the nutritional security issue to a large part of world population.

Biofortification refers to a number of different strategies aimed at the nutritional improvement of food crops, spanning from the agronomic practices and conventional breeding, to the modern biotechnological tools [102] (Table 3). The agronomic methods for biofortification include the application of nutrients to improve the content in minerals, vitamins or bioactive compounds. The typical example of plant biofortification is the improvement of iron, zinc, selenium or iodine content in soil or water or the application of essential micronutrient-based fertilizers to boost the nutritional value of commercial crops [102]. However, in recent years, the definition of biofortification has undergone an extension of its meaning, thus including not only the micronutrient improvement, but also the enhancement of crop nutritional content by including several classes of bioactive compounds and other plant secondary metabolites [103,104,105]. Some agronomical strategies, including the employment of conventional/organic farming methods or reduction of nitrogen fertilization, have been reported to impact on the content of health-promoting secondary metabolites, such as polyphenols [106,107]. Organic farming has been showed positively influencing the levels of polyphenols in vegetables, as in the case of phenolic acids and flavonoids in eggplants [108], cabbage [109] or lettuce [110]. However, the effects of different agronomic management practices on polyphenols levels in edible parts of horticultural crops are not easily predictable [111]. In fact, both conventional or organic farming could concur to nitrogen supply, which is an important parameter influencing either primary and secondary metabolites biosynthesis. However, in the case of polyphenols limited effects have been reported in some cases [112]. In others, a high nitrogen supply in the soil negatively impacted on the flavonoid accumulation in broccoli, basil and tomato [110], or on the anthocyanins content in grapevine berries (Table 3) [113].

Environmental factors and application of stresses can also affect polyphenol accumulation and provide an alternative way for biofortification. Light intensity or UV exposure under a controlled environment can stimulate anthocyanins or flavonoids production, since the biosynthetic pathway can be modulated by light response, as in the case of turnip seedlings [114], dark-purple tea plants [115], or red leaf lettuce (Table 3) [116].

Conventional breeding strategies are the most common way to select agronomic traits carrying nutritional features of consumers’ interest. The genetic improvement can be achieved either by selection of plants with desirable traits for seed and vegetative propagation, or by crossing closely related individuals to produce new hybrids [117]. In both cases, the genetic variability and availability is a necessary condition for the success of the breeding strategy to improve nutritional characteristics. Hence, the selection process starts from the knowledge of the main genetic traits responsible for the synthesis of specific phytonutrients, since a large quantitative variation may occur between cultivated and wild species. In this way, it is possible the introgression of the specific traits from wild to domesticated species. An example of such strategy is represented by the Sun Black tomato variety, which can accumulate anthocyanins in the fruit skin, due to the presence of an introgressed dominant *Aft* (Anthocyanin fruit) gene together with the *atv* (atroviolacea fruit) gene [118,119]. Another example is represented by an eggplant variety derived from some introgression lines (*S. integrifolium*, *S. aethiopicum*, *S. sodomaeum*), which showed enhanced nutritional properties and high levels of polyphenols [120].

Furthermore, new advances in molecular biology, genomics and metabolomics have brought a further contribute in the assisted selection and breeding, and the detection and mapping of the major quantitative trait loci (QTL) is greatly promising for this scope [117].

The biotechnological approach has the advantage to select and directly introduce genetic traits of interest without the strong limitation of compatible genetic background, as in the case of conventional breeding [104]. This approach includes the classical transgenic modifications and the novel breeding technologies (NBTs), which are now rapidly emerging and proposing as the flywheel for the next generation of functional foods. In both cases, a specific plan of metabolic engineering is required, based on a deep scientific knowledge of plant metabolisms, besides the possibility to easily transform and regenerate the desired plant species [121].

Genetic engineering has already showed its potential addressing nutritional requirements, in specific plant organs (i.e., seed, vegetative tissues, fruit) and combining multiple introduced genetic traits without complex and long-term breeding programs (Table 3) [104]. One of the most popular strategies of metabolic engineering include the use of key structural genes of the polyphenol pathway, aiming mostly their over-expression, to stimulate the new biochemical steps that lead to the accumulation of new classes of polyphenols. This strategy resulted in polyphenol content enhancement [122,123,124], even though in some cases with very modest effects [125]. On the other hand, the modulation of polyphenol pathway through the use of regulatory genes encoding transcription factors revealed to be a more effective strategy for polyphenol enhancement in edible crops. Examples of this successfully approach have been reported in apple [126], tomato [127,128], or potato [129].

A second generation of metabolic engineered plant foods include the combination of structural and regulatory genes, a multilevel engineering strategy which led, in some cases, to a high accumulation of polyphenols, enhanced the nutritional potential of relevant crops to reach health benefits [74,130]. Yet, some examples come from tomato, in which the contextual enrichment of high levels of phenolic acids, flavonols, stilbenes, isoflavones or anthocyanins has been realized [74,130]. Anyway, the single or multilevel strategies also allowed to compare the healthy beneficial effects of different classes of phytochemicals within the same food matrix which is an important pre-requisite for the development of tailored crops for specific nutritional requirements.

## 4. The Genome Editing: A Step Forward in Plant Metabolic Engineering

Despite the metabolic engineering approach has offered a new and exciting way of thinking the plant nutritional quality, conventional breeding still remains the most widely used method for crop improvement, although it is labor-intensive and usually takes several years for the screening and identification of genotypes with desirable genetic traits. On the other hand, the use of genetically modified (GM) crops is still largely debated for health and environmental safety concerns, which have greatly restricted the GM use to a small number of available crops (Table 3). More recently, the so-called new breeding technologies (NBTs), which include the genome editing ones, have emerged as very powerful tools for precise modifications of desired genetic traits. The genome editing mediated by transcription activator-like effector nucleases (TALENs) and zinc-finger nucleases (ZFNs) have represented the initial pioneers, although recently the clustered regularly interspaced short palindromic repeats (CRISPR)/Cas system has mainly been preferred and utilized.

In all cases, sequence-specific nucleases (SSNs) recognize target genome sequences and generate double-stranded breaks (DSBs). The endocellular systems (the error-prone non-homologous end joining, NHEJ, or the homologous recombination, HR) repair the DSBs, leading to deletions or insertions, thus causing gene knockout or, in some cases, replacement [131]. Although the NHEJ is the primary and most efficient mechanism of repair in plant cells [132], HR can permit a precise genome repair by using customized template sequences flanked by homologous ends or arms compatible with the DSBs’ site [133].

In the case of zinc finger nucleases (ZFNs), the technology is based on chimeric proteins, composed of an artificial zinc finger DNA binding domain at the N-terminal region and a FokI DNA cleavage domain at the C-terminal region. The zinc finger DNA binding domain can be modified to specifically target the genomic sequence of interest. In fact, since each zinc finger protein recognizes three tandem nucleotides, the manipulation in the order of more zinc fingers enable the recognition and binding to specific sequences. Therefore, the assembly of ZFNs involves the modular design and linking of zinc fingers in a sequence that allow the recognition of the DNA target sequence [134].

TALENs-mediated genome editing is instead based on sequence-specific nucleases consisting of transcription activator-like effectors fused to the catalytic domain of the FokI endonuclease. The DNA-binding domain in TALE monomers is composed of a central repeat domain (CRD) that directs DNA binding [135]. The CRD is formed by tandem repeats, each binding a single nucleotide in the target sequence, a one-to-one pairing that enables TALENs to target the specific sequence [131].

However, recent advances highlighted how the CRISPR/Cas systems can represent a more versatile tool for genome editing in comparison with ZFNs or TALENS [136]. This system takes advantage of a Cas9 endonuclease, composed by a recognition functional domain and a nuclease domain, a guide RNA, formed by the complex of non-coding RNA elements (crRNA) and small trans-encoded RNA elements (i.e., tracrRNA) [137,138,139], which directs the Cas9 endonuclease to the target sequence along with the PAM (protospacer adjacent motif) site. To date, several Cas9 variants are also available, such as Cas9 nickase (nCas9), which is derived from a mutation in native Cas9 domains and has the ability to induce nicks in the genome [135], or dead Cas9 (dCas9), which is basically a catalytically inactive Cas9 [137,140].

dCas9 represents a novel tool for the genetic manipulation through transcriptional alteration since the expression levels of a specific gene can be altered by assembling dCas9 with transcriptional regulators that are recruited to the promoter region. In a similar manner, dCas9 can be fused to epigenetic modulators, such as methylation and deacetylation enzymes. Therefore, CRISPR/dCas9 systems can also be a useful tool for epigenome editing, modulation of chromatin topology and live-cell chromatin imaging in plants. However, suitable transcriptional modulators, accurate study of the target sites, appropriate sgRNA construct design and delivery of the CRISPR/Cas9 system are important requirements for the success of this application [141].

In addition to these Cas9 variants, other CRISPR/Cas systems are now rapidly developing, which might overcome some CRISPR/Cas9 limitations [142]. For example, CRISPR/Cas12 (CRISPR/Cpf1) might enhance the editing efficiency (especially of insertions at the target site) [140,142], CRISPR/Cas13 has an efficiency comparable to RNAi systems [143], or CRISPR/Cas14a, a “miniature” nuclease able to cleave a single-strand DNA, are now being considered for applications in plant species [142,144].

In general, the limitations of the CRISPR/Cas9 system include the availability of suitable plant transformation/regeneration systems. In fact, the most common method of plant transformation is so far mediated by *Agrobacterium* strains, although new delivery systems suitable for protoplasts or biolistic approaches have also been proposed. DNA-free in vitro systems that consist in the direct delivery of a ribonucleoprotein complex, formed by the Cas9 protein and the guide RNA has been recently proposed [127,136,145]. However, these types of delivery show a variable efficiency in different plant species [132], a reason that pushes the necessity of an accurate planning of the plant transformation step.

## 5. Genome Editing Technologies and Nutritional Aspects: A Focus on Polyphenols Biosynthesis Modification

Gene knockouts or insertion mutants produced by genome editing technologies can be useful tools for crop nutritional improvement. The modifications can impact either on anti-nutritional factors or on nutritionally/organoleptic relevant compounds, thus finally influencing the health-promoting properties of food products.

For example, TALENs have been used in potato tubers to knock out the vacuolar invertase gene (*Vinv*), resulting in low levels of undesirable reducing sugars in tubers [146]. A new rice line has been created targeting TALENs on the *OsBADH2* gene, responsible for the biosynthesis of 2-acetyl-1-pyrroline, with the result of restoring the content of this compound, an important component of the fragrance of natural rice mutants [136,147]. TALENs has also been used to knock out the *sterol side chain reductase 2* (*SSR2*) gene, responsible of non-desirable steroidal glycoalkaloids, with a consequent reduction in the levels of chaconine and solanine in new potato lines [148]. TALENs have also been employed for the improvement of starch metabolism in potato tubers [149] or to target the *SlANT1* gene, involved in the anthocyanin biosynthesis in tomato [150].

CRISPR/Cas9 was used to simultaneously target conserved domains in the α-gliadin gene family in wheat and obtain new low-gluten lines [151]. Nutritional quality has also been improved by lowering the content in antinutrient compounds, as the reduction of phytic acid content by inducing mutations on *OsITPK6* gene in rice grain [152], or reducing anti-nutritional proteins by editing the α-amylase/trypsin inhibitor genes in durum wheat [153].

Improved quality by using CRISPR/Cas9 system has been achieved in Camelina sativa seeds producing high-oleic acid levels [154,155], in *Brassica napus*, by editing the fatty acid desaturase 2 gene and improving oleic acid levels [156], in tomatoes, improving the γ-aminobutyric acid content by using a multiplexed CRISPR/Cas9 system on five key genes (*PDS*, *GABATP1*, *GABA-TP2*, *GABA-TP3*, *CAT9*, *SSADH* genes: *phytoene desaturase*, *pyruvate-dependent GABA-transaminase 1*, *pyruvate-dependent GABA-transaminase 2*, *pyruvate-dependent GABA-transaminase 3*, *cationic aminoacid transporter 9* and *succinate semialdehyde dehydrogenase* genes, respectively; [157,158]), or in lycopene-enriched tomatoes by targeting *SGR1*, *LCY-E*, *Blc*, *LCY-B1* and *LCY-B2* genes (*stay-green 1*, *lycopene δ-cyclase*, *beta-lycopene cyclase*, *lycopene β-cyclase 1* and *2*, respectively) [159].

CRISPR/Cas9 system has been studied and tested also for polyphenol biosynthesis, in some cases to test the blockage at some levels of the pathway, that can give an easy colored proof-of-concept of its correct functioning, and eventually to provide a demonstration of the application of the system [160,161,162] (Table 4). For example, CRISPR/Cas9 targeting the carrot *flavanone-3-hydroxylase* (*F3H*) gene have been tested for blocking the anthocyanin biosynthesis in a model of purple-colored calli [163], torenia [161], petunia [164] and black rice [165].

In the specific case of polyphenol content improvement, the CRISPR/Cas9 system has been employed to reduce/knockout the function of negative regulators of the phenylpropanoid/flavonoid pathway or vice-versa by trying to enhance the function of genes expression activators implicated in polyphenol biosynthesis. As an example, the regulatory *SlMYB12* gene knocking out have led to an accumulation of flavonoids in pink-colored tomato fruits [166]. *SlAN2* tomato mutants, exhibiting anthocyanin pigmentation and accumulation similarly to the ‘Indigo Rose’ variety, has been generated using CRISPR/Cas9 targeted on the gene sequence encoding the AN2 MYB transcription factor [167]. CRISPR/Cas9 has also been employed to induce mutations on the regulatory *BnTT8* gene. The mutants showed a suppressed phenylpropanoid/flavonoid pathway and pro-anthocyanidin deposition in the seed coat and a desirable trait of yellow-seeds [168]. On the other hand, Čermák and co-workers [150] have delivered a CRISPR/Cas9 together with a geminivirus-based replicon system to target the *SlANT1* gene and introduce upstream to its sequence a constitutive promoter, exploiting the HR mechanism of repair and thus developing new edited tomato lines that accumulate anthocyanins in the whole plants, including the fruits. Furthermore, in this study, the targeted DNA insertions were observed in segregating population with purple phenotypes, confirming the hereditability of the new mutated alleles. A similar approach was employed using CRISPR/Cpf1 in tomato to enhance the expression of the *SlANT1* gene and resulting in anthocyanin-pigmented tomato plants [169], with similar patterns of segregation in the progeny as observed by Čermák and co-workers [150]. In another work, the HR mechanism of repair has been exploited to repristinate the anthocyanin pigmentation in tomato seedlings following a CRISPR/Cas9-induced deletion on the *SlDFR* target gene [170].

Other studies targeted the polyphenol oxidase (PPO), one of the polyphenols degrading-enzymes in fruit and vegetable, resulting in non-browning mushrooms and potatoes [171,172].

All these examples represent new and stimulating approaches to preserve or improve the nutritional value of crop species (Table 4).

**Table 4:** Examples of genome editing systems for the study, blockage, enrichment or preservation of the polyphenol content in plant models or crop species.

Model or Species	Target Gene	Genome Editing System	Effect	References
Japanese morning glory (*Ipomoea [Pharbitis] nil*)	*Dihydroflavonol-* *4-reductase-B* (*InDFR-B*)	CRISPR/Cas9	White flower mutants	[160]
Purple-colored calli of carrot (*Daucus carota* L.)	*Flavanone-3-hydroxylase* (*DcF3H*)	CRISPR/Cas9	Block of anthocyanin biosynthesis, resulting in non-purple-colored calli	[163]
Torenia plant (*Torenia fourneri* L.)	*Flavanone-3-hydroxylase* (*TfF3H*)	CRISPR/Cas9	Block of anthocyanin biosynthesis, resulting in pale blue flowers	[160]
Petunia (*Petunia hybrida* cv. Madness Midnight)	*Flavanone-3-hydroxylase* (*PhF3H*)	CRISPR/Cas9	Modified, pale purplish flower color	[164]
Sage (*Salvia miltiorrhiza*) hairy roots	*Rosmarinic acid synthase* (*SmRAS*)	CRISPR/Cas9	Decreased content of phenolic acids, including rosmarinic acid and lithospermic acid	[173]
Black rice (*Oryza sativa* L. *cv* Heugseonchal and Sinmyungh)	*Flavanone-3-hydroxylase, Dihydroflavonol-* *4-reductase, Leucoanthocyanidins dioxygenase (OsF3′H, OsDFR and OsLDOX)*	CRISPR/Cas9	Reduction of anthocyanin accumulation in mutant lines	[165]
Japanese gentian (*cv* ‘Albireo’, *Gentiana* *triflora* × *Gentiana scabra*)	*Anthocyanin- related glutathione S-transferase* (*GST*)	CRISPR/Cas9	Reduction of anthocyanin content	[162]
Tomato (*Solanum lycopersicum* L.)	*Dihydroflavonol-* *4-reductase (SlDFR)*	CRISPR/Cas9	Reduction of anthocyanin content in seedlings	[170]
Pomegranate (*Punica granatum* L.) hairy roots	*UDP-dependent glycosyltransferases* (*PgUGT84A23* and *PgUGT84A24*)	CRISPR/Cas9	Changes in galloyl-glucose conjugates and derivatives	[174]
Rapeseed (*Brassica napus* L.)	*TRANSPARENT TESTA 8* (*BnTT8*)	CRISPR/Cas9	Yellow seeds with improved oil and protein contents, block of pro-anthocyanidin deposition in seed coat	[168]
Tomato (*Solanum lycopersicum* L.)	*Anthocyanin mutant 1* (*SlANT1*)	TALENs CRISPR/Cas9 CRISPR/Cpf1	Anthocyanin accumulation in the whole tomato plant	[150,169]
Tomato (*Solanum lycopersicum* L.)	*R2R3MYB transcription factor 12* (*SlMYB12*)	CRISPR/Cas9	Accumulation of flavonoids in pink-colored tomato fruits	[166]
Tomato (*Solanum lycopersicum* L.)	*Anthocyanin 2* (*SlAN2*)	CRISPR/Cas9	Anthocyanin pigmentation in tomato fruits	[167]
Grapevine (*Vitis vinifera* L. rootstock 101-14)	*Trans-Acting Small-interfering locus4 (VvTAS4) and R2R3MYB transcription factor A5/6/7 (VvMYBA5/6/7)*	CRISPR/Cas9	Lack of visible pigment phenotypes in edited plants	[175]
Mushroom (*Agaricus bisporus*) Potato (*Solanum tuberosum* L.)	*Polyphenol oxidase* (*PPO*)	CRISPR/Cas9	Reduced fruit browning	[171,172]

## 6. Conclusions and Perspectives

In this review, we focused on the benefits that polyphenols bring to gut health, with special attention to their antioxidant and anti-inflammatory activities that can influence the immune-modulatory response at the basis of many intestinal diseases. Nutritional interventions for the prevention of these pathological processes represent an important hallmark for preventive nutrition. However, daily polyphenol intake is considered suboptimal for most people living in Western countries. Requests to change the nutritional habits and increase the consumption of fruits and vegetables are multiplying to address this goal and in general the nutritional security issue for Western countries populations. In this context, two possible strategies for polyphenols daily intake increase can be utilized. The first refers to the (re)discovery of under-utilized niche species that accumulate high levels of secondary metabolites in edible parts of the plant. Some examples are listed in Table 1. However, robust characterization of the main classes of secondary metabolites still lacks for many of these species and future efforts have to be undertaken to fill this gap. A second interesting strategy to increase the content of healthy components in major crops refers to all the biofortification strategies so far developed. Table 3 of this review reports about all the main traditional or innovative techniques to address this goal with limitations or strength points for each one. Among the conventional ones, breeding techniques require a long time for the selection of specific genetic traits of interest and their introgression in cultivated varieties. Agronomic practices can influence either yield and quality of crops, but results so far obtained on the polyphenols content look controversial. Stress application can be an exciting option, but it requires great attention to select those impacting the least possible on the final yield and quality of fresh products.

Among biofortification strategies more recently proposed and tested for polyphenols improvement, the multi-level metabolic engineering approach is an elegant strategy to finely regulate and induce new target genes, but restrictive regulatory policies in some countries (e.g., Europe) and a limited public acceptance have so far greatly limited their success.

New breeding technologies are emerging as possible alternatives to overcome the limitations of GMOs. However, given the wide versatility of genome-editing applications, it will be essential to select the most suitable systems for each plant species and intervention type (gene knockout/insertion). However, despite the many technical challenges (e.g., efficiency of the genome editing systems, efficiency of plant transformation, selection of transgenic-free mutant lines) still to be addressed, other important issues regarding the general public’s acceptance and the adoption of more permissive regulatory policies must be considered and settled before the wide application of these techniques in agriculture and improved crop varieties onto the market.

## Figures and Tables

**Table 3:** Schematic summary of common biofortification methods for the improvement of polyphenol content in plant foods.

Biofortification Method		Pros	Cons
Conventional approaches	Agronomic practices/ fertilization strategies	Free choice of farming practices; reduction of nitrogen supply	High variability in different harvesting seasons Not predictable/limited effects for the biofortification scopes
	Exposition to stresses (e.g., light-UV treatments)	Natural stimulation of polyphenol biosynthetic pathway	Treatments need highly controlled conditions Stress application can impact on yield and quality of fresh products.
	Conventional breeding programs	Selection of genetic trait of interest	Reduced genetic variability needs to be considered for each species Long time required for screening and obtaining the phenotype/genotype of interest
Biotechnological approaches	Transgenic methods	Direct insertion or knock out of specific genetic traits; Shorter period of time to obtain new improved lines	Restrictive regulatory policies in some countries (e.g., Europe); Limited public acceptance; Still debated for human health and environment safety concerns; Some species are recalcitrant to genetic transformation/regeneration.
	Genome editing technologies	Direct insertion or knock out of specific genetic traits; Shorter period of time to obtain new improved lines	Restrictive regulatory policies in some countries (e.g., Europe) Debate about traceability of the plants/products obtained with these techniques (possibility to (un)distinguish from natural occurring mutations); Some species are recalcitrant to genetic transformation/regeneration.
